# Imputing HIV treatment start dates from routine laboratory data in South Africa: a validation study

**DOI:** 10.1186/s12913-016-1940-2

**Published:** 2017-01-17

**Authors:** Mhairi Maskew, Jacob Bor, Cheryl Hendrickson, William MacLeod, Till Bärnighausen, Deenan Pillay, Ian Sanne, Sergio Carmona, Wendy Stevens, Matthew P Fox

**Affiliations:** 1 Heath Economics and Epidemiology Research Office, Department of Internal Medicine, School of Clinical Medicine, Faculty of Health Sciences, University of the Witwatersrand, Johannesburg, South Africa; 2 Department of Global Health, Boston University School of Public Health, Boston, MA USA; 3 Africa Health Research Institute, Somkhele, South Africa; 4 Institute of Public Health, University of Heidelberg, Heidelberg, Germany; 5 Division of Infection and Immunity, University College London, London, UK; 6 Department of Internal Medicine, Clinical HIV Research Unit, School of Clinical Medicine, Faculty of Health Sciences, University of the Witwatersrand, Johannesburg, South Africa; 7 National Health Laboratory Service, Johannesburg, South Africa; 8 Department of Molecular Medicine and Hematology, University of the Witwatersrand, Johannesburg, South Africa; 9 Department of Epidemiology, Boston University School of Public Health, Boston, MA USA

**Keywords:** Health systems, Monitoring and evaluation, Resource-limited settings, Missing data, Imputation, Validation, Laboratory, HIV/AIDS, Antiretroviral therapy, South Africa, Chronic disease management

## Abstract

**Background:**

Poor clinical record keeping hinders health systems monitoring and patient care in many low resource settings. We develop and validate a novel method to impute dates of antiretroviral treatment (ART) initiation from routine laboratory data in South Africa’s public sector HIV program. This method will enable monitoring of the national ART program using real-time laboratory data, avoiding the error potential of chart review.

**Methods:**

We developed an algorithm to impute ART start dates based on the date of a patient’s “ART workup”, i.e. the laboratory tests used to determine treatment readiness in national guidelines, and the time from ART workup to initiation based on clinical protocols (21 days). To validate the algorithm, we analyzed data from two large clinical HIV cohorts: Hlabisa HIV Treatment and Care Programme in rural KwaZulu-Natal; and Right to Care Cohort in urban Gauteng. Both cohorts contain known ART initiation dates and laboratory results imported directly from the National Health Laboratory Service. We assessed median time from ART workup to ART initiation and calculated sensitivity (SE), specificity (SP), positive predictive value (PPV), and negative predictive value (NPV) of our imputed start date vs. the true start date within a 6 month window. Heterogeneity was assessed across individual clinics and over time.

**Results:**

We analyzed data from over 80,000 HIV-positive adults. Among patients who had a workup and initiated ART, median time to initiation was 16 days (IQR 7,31) in Hlabisa and 21 (IQR 8,43) in RTC cohort. Among patients with known ART start dates, SE of the imputed start date was 83% in Hlabisa and 88% in RTC, indicating this method accurately predicts ART start dates for about 85% of all ART initiators. In Hlabisa, PPV was 95%, indicating that for patients with a lab workup, true start dates were predicted with high accuracy. SP (100%) and NPV (92%) were also very high.

**Conclusions:**

Routine laboratory data can be used to infer ART initiation dates in South Africa’s public sector. Where care is provided based on protocols, laboratory data can be used to monitor health systems performance and improve accuracy and completeness of clinical records.

**Electronic supplementary material:**

The online version of this article (doi:10.1186/s12913-016-1940-2) contains supplementary material, which is available to authorized users.

## Background

In many low- and middle-resource settings, clinical records are often incomplete, missing, and/or poorly archived [1]. Where patient records are hand-written, implementation and maintenance of electronic records can be expensive and still not impervious to error [2–4]. Accurate clinical record keeping is increasingly important to clinical care and appropriate monitoring and evaluation in such settings [5]. Many developing country health systems were set up to provide maternal services, vaccinations, and acute care for injury and infections. However, the rising global burden of chronic disease requires health systems to manage patients’ health longitudinally, a challenge in the absence of accurate clinical information. Information gaps also make it difficult to monitor large health care programs and allocate resources optimally [6]. Improved record keeping from treatment sites or cohorts is particularly challenging in settings of high patient mobility across sites, providers, and sectors (public/private), and yet it is precisely in such settings that complete and accurate records are critical to the coordination of patient care [1, 3, 7].

For many diseases, laboratory testing is a meticulously specified element of clinical protocols, as it is the basis for many diagnostic and treatment decisions in low-resource settings. Laboratory tests are often conducted off-site at central laboratories and test results may be available directly from the laboratories, bypassing two steps prone to human error: manual entry of data into patient charts and manual transcription of chart information into electronic databases. Where data from routine laboratory tests are systematically available, such information may be used to impute missing data in clinical records.

As with other chronic diseases, HIV/AIDS requires lifelong clinical management and decision-making guided by routine laboratory monitoring. South Africa has the largest number of people living with HIV/AIDS worldwide, with 7 million adults living with HIV in 2015 [8] and approximately half of these receiving antiretroviral therapy [9]. According to South African national ART treatment guidelines [10–12], patients initiating ART should have certain laboratory tests conducted prior to starting treatment, hereafter referred to as “ART workup”. Clinicians use this combination of blood tests to assess liver function, anemia, and other factors. This allows the clinician to accurately stage the patient’s disease, treat any co-morbid conditions, and guide the choice of treatment regimen.

**Fig. 1:**
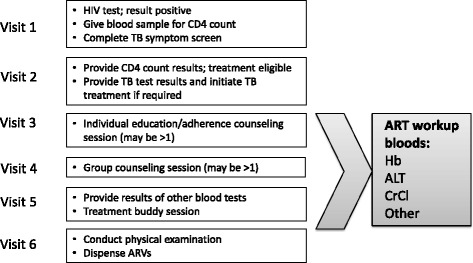
Visit schedule prior to ART initiation under treatment protocols during the study period

We propose using routine laboratory data from South Africa’s National Health Laboratory Service (NHLS) to impute dates of antiretroviral treatment (ART) initiation among patients in South Africa’s public sector HIV care and treatment program. South Africa has detailed national guidelines for initiation of HIV care and treatment [10–12] (Fig. 1). Following a positive result from an HIV test, a CD4 count test is done to determine immune function and, historically, eligibility for initiation of ART. As of September 2016, all patients are eligible for ART at diagnosis, but CD4 counts are still done at enrollment. Once eligibility and intent to initiate is established, the ART workup blood tests are conducted to guide regimen choice. Patients then attend approximately three once-weekly counseling and preparatory sessions before initiating ART (under guidelines during the study period). We thus proposed to impute ART start dates by identifying ART workup dates and adding 21 days. To determine the success of the approach we assessed its validity with respect to sensitivity, specificity, positive predictive value, and negative predictive value. Sensitivity refers to the proportion of true ART start dates accurately identified using our approach. Conversely, positive predictive value captures the proportion of imputed start dates that accurately identify a true start date. Specificity and negative predictive value describes the accuracy of our approach in identifying non-initiators.

NHLS provides laboratory services to all public-sector facilities in South Africa (the province of KwaZulu-Natal joined NHLS in 2010), including all laboratory testing required for ART monitoring with over 26 million CD4 counts and 13 million viral loads resulted between 2004 and 2015. If successful, our imputation approach has the potential to improve the capabilities of laboratory data to monitor and evaluate South Africa’s national HIV care and treatment program.

## Methods

### Data sources and study population

To assess the feasibility and validity of imputing ART start dates from ART workup dates, we analyzed data from two large clinical research cohorts with known dates of ART initiation that are already integrated with the NHLS database [13, 14]. The Hlabisa HIV Treatment and Care Programme Cohort is a rural public sector ART program that includes all HIV patients initiating ART at 17 primary health care clinics and one sub-district hospital in rural Hlabisa sub-district of KwaZulu-Natal province since mid-2004. Since 1 January 2007, the Hlabisa Cohort has systematically collected data on all HIV patients receiving pre-ART care, including those who never go on to initiate ART, one of the few large South African cohorts to do so. Chart-based data on dates of ART initiation were available in the Hlabisa Cohort through the end of 2012. The Right to Care (RTC) Clinical Cohort includes all HIV patients initiating ART at seven RTC-supported primary health care clinics and one provincial hospital in urban and peri-urban settings in Gauteng province. Patients do not routinely enter the database until they initiate ART, at which point data prior to ART initiation is entered retrospectively. All data, including demographic, clinical conditions, laboratory test results and medications (ART and non-ART related) are entered into an electronic patient management and decision support system (TherapyEdge-HIV™). The selection of these cohorts reflects the wide diversity of settings in which care is provided in South Africa’s public sector: urban vs. rural, hospital-based vs. clinic-based. Ethical clearance to analyze data from these cohorts was granted by the Human Research Ethics Committee of the University of the Witwatersrand as well as the Boston University Institutional Review Board.

For both cohorts, data on laboratory tests — including CD4 counts, viral loads, and the ART work-up tests — are electronically integrated directly from the NHLS as results become available, rather than being captured retrospectively from charts or paper records. As all laboratory data in the NHLS database (which contains no information on date of ART initiation) is also in the clinical databases of the Hlabisa and Right to Care cohorts, which do contain data on date of ART initiation, we validated our method (described below) for imputing ART initiation date from laboratory data in each of these clinical cohorts. Our analysis included all patients in the Hlabisa Cohort who had an ART workup or initiated ART between 1 January 2007 and 31 December 2012; and all patients in the RTC cohort who initiated ART between 1 January 2005 and 31 December 2014. Since we were interested in patients’ date of ART initiation, we excluded patients identified as having transferred into the cohorts after having previously initiated ART elsewhere.

### Imputed ART initiation dates using laboratory data

During the period 2004-2014, guidelines required, at a minimum, testing of hemoglobin (Hb) and alanine transaminase (ALT) as part of the ART workup. We thus defined an ART workup date as the date of a patient’s first documented Hb or ALT among patients with a CD4 count in the previous 12 months prior to, or in the month following, the ART workup date. Patients without a previous CD4 count were not coded as having had an ART workup because they may have received Hb or ALT tests as part of investigations for other clinical conditions (e.g. anemia or liver dysfunction respectively). Though the frequency of Hb/ALT without a prior CD4 is relatively low in the HIV cohorts we analyzed, it may be more common in other settings; including CD4 counts enables application of our approach to settings (such as the full NHLS database) where patients' HIV status is not always known. The requirement of a CD4 count may reduce the sensitivity of our approach, as some patients are initiated on ART without a prior recorded CD4 count; we thus present sensitivity analyses excluding the prior 12-month CD4 criterion. As described previously, the three once-weekly counseling sessions between ART workup and ART initiation generate an interval of approximately three weeks between an ART workup and ART initiation (Fig. 1). Therefore, applying this assumption, we imputed ART start dates by adding 21 days to the date of a patient’s lab work-up.

### Known dates of ART initiation in the Hlabisa and Right to Care cohorts

The Hlabisa and Right to Care cohorts contain known dates of ART initiation that are collected systematically from electronic clinical records. Known date of initiation is defined as the earliest date when a triple-drug ART regimen was dispensed. Dates of initiation are recorded in the databases as the date when pills are first dispensed based on prescription chart data. This information is then extracted from clinical records by trained data clerks and input into the RTC and Hlabisa databases. Although it is possible that in some cases, the date of initiation was entered incorrectly, because patients return for care weekly and then monthly after starting ART, it is unlikely that dates of initiation were more than a week or two off.

### Validation of imputed ART start dates

Though ART guidelines are applied nationally, there could be variation in degree of adherence to them due to individual facility circumstance and prevailing clinical protocols. Therefore, we undertook two validation exercises to assess the performance of our imputed ART start dates relative to actual known dates of ART initiation in the Hlabisa and RTC cohorts. First, to test the assumption of 21-days between ART workup and ART initiation that we used to impute ART start date created with the laboratory data, we set out to describe the distribution of times from ART workup to known ART initiation in the clinical cohorts. Restricting our sample to patients with both an ART workup date and a known ART start date, we calculated the number of days from the ART workup date to the known ART start date. Stratifying by cohort, we described the cumulative density function (CDF) of the distribution graphically and calculated the median and other percentiles of the distribution. Additionally, we assessed variation in the median time from work-up to ART initiation across different clinic sites and over time.

Second, we evaluated the performance of our algorithm for imputed ART start dates using traditional test performance criteria. Using the known dates of ART initiation as the gold standard, we calculated the sensitivity, specificity, positive predictive value, and negative predictive value of our imputed ART start dates. We consider our imputed start date as a “match” to the known ART start date if the imputed start date was within a six-month window period of the known ART start date. Six months was selected as the initial cutoff period as clinical care in these settings mandates a CD4 count result is only valid for determining ART eligibility for a period of six months from the test date after which it must be repeated. Based on the shape of the distribution assessed in the first validation exercise (the long right tail), an asymmetric window was chosen. The six-month window period to determine a match (matching window period) began on the date of the ART workup (equivalent to imputed start date *minus* 21 days) and extended six months later (equivalent to imputed start date *plus* 161 days).

Sensitivity of the imputation approach was defined as the probability that a patient who truly initiated ART had an ART workup in the matching window period and thus had an accurate imputed ART start date. Sensitivity can be interpreted as the proportion of ART start dates that would be accurately identified using information only on ART laboratory work-up dates. We estimated sensitivity separately for each of the cohorts and across clinics and years within each cohort. As complete pre-ART data are necessary to calculate positive as well as negative predictive value and specificity, we limited these analyses to the Hlabisa cohort. Positive predictive value (PPV) of the imputation approach was defined as the proportion of patients with an ART workup that truly initiated ART within the matching window period. Specificity of the imputation approach was defined as the probability that a patient who truly did not initiate ART also did not have an ART workup. Because patients who did not initiate ART had no date from which to estimate a six-month window, specificity was assessed as the probability of never having had a work-up conditional on never having initiated ART by the end of follow-up. Similarly, negative predictive value (NPV) was defined as the probability that a patient who was diagnosed with HIV and had a CD4 count but ﻿never had an ART workup also never initiated ART during follow-up.

## Results

We analyzed data on 57,401 patients in the Hlabisa Cohort; 38% (*n =* 21,766) of these had a documented ART initiation date in the clinical database while 33% (*n =* 18,836) had a documented ART workup. An additional 493 (0.9%) had an Hb/ALT result without a CD4 count. Currently, the RTC cohort contains data on over 133,000 patients, of whom 102,070 have initiated ART. Of these, we analyzed the 69,283 (68%) with a documented ART initiation date, of whom 63,914 (92%) had a documented ART workup and an additional 1153 (1.6%) had an Hb/ALT without a CD4 count. The cohorts were similar with respect to age and gender distribution while those in the RTC cohort initiated at slightly lower CD4 counts (median 123 vs. 152 cells/mm^3^) compared to those in the Hlabisa cohort (Table 1).

**Table 1:** Demographic and clinical features of 90,281 patients at ART initiation in the Hlabisa and Right to Care cohorts in South Africa

		Hlabisa Cohort^a^	RTC Cohort^b^
		(*n =* 21,766)	(*n =* 69,283)
Gender	% male	7421 (34.1%)	25902 (37.4%)
Age at initiation (years)	<18	2092 (9.6%)	3390 (4.9%)
	18-29	6369 (29.3%)	15470 (22.3%)
	30-39	7294 (33.5%)	28286 (40.8%)
	40-49	3880 (17.8%)	15334 (22.1%)
	> = 50	2125 (9.8%)	6803 (9.8%)
	Median (IQR)	33 (26, 41)	35 (29, 42)
CD4 count (cells/mm^3^) at initiation	0-49	3282 (16.1%)	15228 (22.0%)
	50-99	3220 (15.8%)	10699 (15.4%)
	100-199	7567 (37.0%)	19795 (28.6%)
	200-349	4818 (23.6%)	11346 (16.4%)
	> = 350	1540 (7.5%)	4178 (6.0%)
	Missing	1339 (6.2%)	8037 (11.6%)
	Median (IQR)	152 (78, 226)	123 (50, 201)
Year of ART initiation	2005	n/a	2975 (4.3%)
	2006	n/a	5339 (7.7%)
	2007	1963 (9.0%)	6530 (9.4%)
	2008	3467 (15.9%)	8612 (12.4%)
	2009	3223 (14.8%)	10546 (15.2%)
	2010	3723 (17.1%)	11036 (15.9%)
	2011	4607 (21.2%)	8325 (12.0%)
	2012	4783 (22.0%)	6744 (9.7%)
	2013	n/a	5457 (7.9%)
	2014	n/a	3719 (5.4%)

^a^Hlabisa Cohort includes all HIV patients with a first CD4 count in care between 1 January 2007 and 31 December 2012 at 17 clinics and one sub-district hospital in Hlabisa, KwaZulu-Natal. Data in this table are restricted to patients who initiated ART. Hlabisa Cohort also includes patients who presented for care but never initiated ART. Total sample size in the Hlabisa Cohort was 57,401 of whom 21,766 initiated ART

^b^RTC Cohort includes all patients who initiated ART between 1 Jan 2005 and 31 December 2014 at 8 sites supported by Right to Care in Gauteng Province

**Fig. 2:**
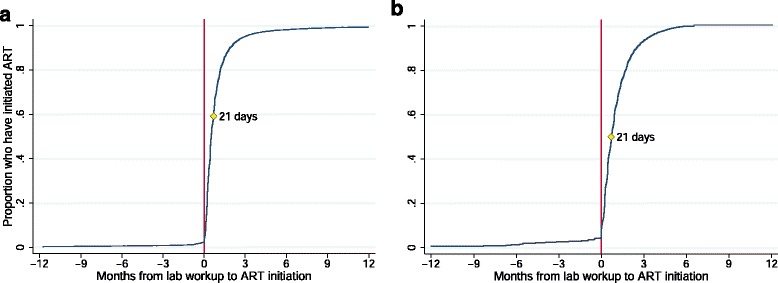
Time from ART workup date to known ART initiation date Fig. 2**a** displays the cumulative distribution for Hlabisa; Fig. 2**b** displays the cumulative distribution for Right to Care. In the Hlabisa Cohort, 2.3% of patients with an ART workup had initiation dates more than 182 days later or had not initiated by the end of follow-up. As this population was not observed in the RTC cohort, patients initiating more than 182 days or not at all are excluded from both plots

We calculated the number of days from the ART workup date to the known date of ART initiation for each patient (Fig. 2). Very few patients (2.5% in the Hlabisa Cohort, 4.4% in the RTC cohort) had their first recorded ART workup after ART initiation. These patients may have transferred into care and not been identified as such in the cohort dataset. The vast majority of patients who initiated ART did so after the workup date and 95% of patients in both the Hlabisa Cohort and RTC cohorts who initiated ART did so within 91 days after the ART workup. The median interval from ART workup to ART initiation was 16 days (IQR 7 – 31) in the Hlabisa cohort and 21 days (IQR 8 – 43) in the RTC cohort.

Median times from lab workup to ART initiation in the two cohorts were not only very similar to each other but also remarkably close to our hypothesized interval of 21 days from ART workup to initiation based on the initiation process outlined in national guidelines. While this suggests that, in general, sites implement procedures as outlined in the guidelines, practices could vary considerably by facility. We thus stratified this analysis by each of the facilities contributing data towards the larger Hlabisa and RTC cohorts. While there was some variation in the median number of days from ART workup to initiation between certain sites and we could not conclude there was no clinic-level variation, the overall consistency of the interval between workup and ART start dates by site was reassuring (Additional file 1: Table S1).

We also considered that there might be an association between calendar year and time from workup to treatment initiation as treatment guidelines have evolved somewhat over time and clinics may have become more efficient at initiating eligible patients as the rollout of the treatment program matured. We stratified the median number of days to initiation by calendar year and noted a decreasing trend in the number of days to initiation over our period of study (Additional file 1: Table S2). This decrease in the interval to initiation was remarkably similar for both cohorts and consistent with known programmatic changes in the treatment initiation guidelines towards faster ART initiation. Of note, 2008 PMTCT guidelines mandated that all pregnant women should be fast-tracked on ART [15], though no exact timeline was stipulated and clinic-level implementation may have been formalized somewhat later. In 2010, updated guidelines specified that pregnant women, patients with drug-resistant tuberculosis, and patients with nadir CD4 cell counts <100 cells/mm^3^ should be fast-tracked onto ART within 2 weeks of determining eligibility [11]. In 2012, fast-tracking was further expanded to include initiation within 2 weeks for those with CD4 counts < 350 cells and same-day initiation for those with CD4 counts <200 and all pregnant women regardless of CD4 count (Communication, National Department of Health, 2012). Thus, while workup date *plus* 21 days is a reasonable estimate for dates of initiation historically, analyses of patients initiating ART in more recent years and in the future might consider downward revisions to the 21-day interval. We note that differences of 2-3 weeks are unlikely to affect most analyses, and indeed our estimates of sensitivity, specificity, and positive and negative predictive values were virtually unchanged when imputing using the estimated annual median time to initiation in place of the 21-days value.

Having established 21-days as a reasonable estimate of time to initiation for patients with both workup dates and ART start dates, we turned to an assessment of the validity of using workup date *plus* 21 days to impute ART start dates. We estimated the sensitivity of our imputed start date for each of the cohorts based on whether the imputed date was considered a “match” to the known start date as defined above. Our results demonstrated high sensitivity of the imputed date. Among those with known ART start dates, very similar proportions (82.6% in Hlabisa and 88.2% in RTC) had a matched imputed start date (Table 2), indicating this imputation method will correctly estimate the ART start date as defined within a 6 month window period for roughly 85% of those who initiated ART. The sensitivity of our imputation approach was consistently high over time ranging from 78 to 93% over the 10 calendar years evaluated (Additional file 1: Table S2), and across facilities, ranging from 77 to 97% in all but two sites (Additional file 1: Table S1).

**Table 2:** Sensitivity of imputation method in Hlabisa and RTC cohorts

*Hlabisa Cohort: Sensitivity*		Truly initiated ART = yes	
		HGB/ALT + CD4	HGB/ALT only
ART workup in 6mo prior to known ART start date?	Yes	17,970	18,393
	No	3,796	3,373
	Total	21,766	21,766
	Sensitivity	82.6%	84.5%
*RTC Cohort Sensitivity*		Truly initiated ART = yes	
		HGB/ALT + CD4	HGB/ALT only
ART workup in 6mo prior to known ART start date?	Yes	61,105	61,632
	No	8,178	7,651
	Total	69,283	69,283
	Sensitivity	88.2%	89.0%

Next, we restricted the analysis to the Hlabisa cohort and evaluated the PPV, NPV, and specificity (Table 3). Among all patients with an ART workup — including those that never initiated — the probability that a patient went on to initiate ART within the six-month window was PPV= 95.4%, implying that fewer than 5% of patients with an ART workup would be falsely identified as initiating ART using our imputation strategy. Specificity and NPV were also both high (99.8% and 92.2% respectively). Sensitivity and NPV increased slightly after removing the requirement of a CD4 count in the previous 12 months from the definition of an ART workup (Tables 2 and 3, rightmost column).

**Table 3:** Sensitivity, specificity, positive and negative predictive values of imputation method in the Hlabisa cohort

*Specificity*		Truly initiated ART = no	
		HGB/ALT + CD4	HGB/ALT only
Ever had an ART workup?	Yes	79	84
	No	35,556	35,551
	Total	35,635	35,635
	Specificity	99.8%	99.8%
*Positive Predictive Value (PPV)*		Had ART workup = yes	
		HGB/ALT + CD4	HGB/ALT only
Known ART start date in 6mo after ART workup date?	Yes	17,970	18,393
	No	866	963
	Total	18,836	19,329
	PPV	95.4%	95.2%
*Negative Predictive Value (NPV)*		Had ART workup = no	
		HGB/ALT + CD4	HGB/ALT only
Ever initiated ART?	Yes	3009	2,521
	No	35,556	35,551
	Total	38,565	38,072
	NPV	92.2%	93.4%

Table displays estimates in separate columns for our primary definition of ART workup: a patient’s first haemoglobin or ALT occurring up to 12 months after or one month before a CD4 count – and our secondary definition: first haemoglobin or ALT, regardless of CD4 count. For sensitivity and positive predictive value, an imputed ART start date and known start date were considered to “match” if the imputed date occurred in the interval 5 months and 1 week prior to and up to 3 weeks after the known ART start date. Because we imputed by adding 21 days to the date of the lab workup, a “match” occurred if the workup date was in the six months prior to known start date. For specificity and negative predictive value, there was no reference date – of initiation or lab workup – with which to define a six-month interval. Therefore, we report specificity and NPV for whether a patient *ever* had workup or initiated ART, respectively. We note that these are lower bounds for sensitivity and NPV, which would be higher were we to assign fake dates of initiation or workup to patients without them. Data are presented for Hlabisa only, as pre-ART data were not systematically collected in the RTC cohort

As highlighted previously, we used a guideline-driven initial window period of 6 months to define a “matching” period for the imputed initiation dates. However, this period is arbitrary, and as we show in Fig. 2, the vast majority of patients initiated within a much shorter window. We thus created several alternate matching window periods varying from 1 month to 9 months and estimated sensitivity of the imputation approach with these changes in window period (Additional file 1: Table S3). Sensitivity dropped to about 60% when the window was restricted to one month but was over 80% at 3, 6, and 9 months.

## Discussion

Our analysis has a number of implications. The results provide guidance on whether the date of an ART workup can be used to infer dates of ART initiation in South Africa, and with what degree of certainty. We demonstrate high sensitivity and high positive predictive value of this approach, and performance appears to be consistent across diverse geographic and facility settings and over time. Laboratory-imputed initiation dates could be used as part of an effort to create a national clinical cohort from laboratory databases such as the NHLS (i) to monitor and evaluate South Africa’s ART program and (ii) to improve clinical records, patient care and accountability at all levels of the healthcare system. The implications for the evaluation of South Africa’s national program could be significant. The ability to impute dates of initiation within the national NHLS database will enable evaluation of key aspects of the national program, providing strong evidence (in a resource efficient manner) beyond what existing surveillance systems and cohort estimates have been able to do to date. These data could also be used to monitor facility performance and to improve accountability, although vigilance is required against the possibility of adverse behavioral consequences (e.g., conducting unnecessary Hb/ALT tests to boost numbers).

Laboratory testing is used routinely in determining HIV disease progression a nd assessing ART eligibility (historically), determiningART regimen, and monitoring patients on therapy in many southern African countries, though the specific protocols for how laboratory testing is used vary across countries and coverage is uneven [16–23]. Although our approach — imputing clinical data using laboratory results — may have broad applicability, the specific imputation algorithms used may differ in other countries and will have to be validated in those contexts. Our analysis yields a validated algorithm for South Africa and a proof of concept that could be applied in other countries, particularly as laboratory infrastructure improves.

Our findings also have implications for understanding the continuum of HIV care and treatment. Although significant losses have been described between HIV testing and ART initiation both in the Hlabisa cohort [24] and elsewhere [25], our results on positive predictive value imply that the losses from date of ART work-up to ART initiation are modest. This implies that losses to initiation are occurring primarily within the first few clinic visits, between HIV testing and collection of workup bloods. Future interventions could target these early stages in the cascade of care, where most attrition occurs.

The methods described here to leverage routinely collected laboratory data may have other applications. First, improving the accuracy and completeness of clinical records may be an important and cost-effective step in overcoming obstacles to effective chronic disease management in low resource settings. Integration of laboratory data into clinical records could help manage highly mobile patient populations. Second, this methodology may offer several opportunities to enhance existing monitoring and evaluation strategies. Laboratory data could be used to triangulate against aggregate (e.g. district-level) information on new ART initiates based on clinic reporting. Because sensitivity is lower than PPV, the number of ART workups is expected to be lower than the number of ART initiators. However, the number of ART initiators can be estimated easily by multiplying the number of workups in a given population by a factor equal to PPV/sensitivity, equal to 1.15 in the Hlabisa cohort.^1^ Of course, the generalizability of this adjustment factor depends on the generalizability of our sensitivity and PPV estimates. In settings where nationally complete registration data is not available, linkages between various sources of “big” data can significantly improve monitoring disease burden and health system response. South Africa has a national vital registration system, the National Population Register, and linkages between this register and several HIV cohorts have successfully improved ascertainment of mortality among those on ART [26, 27]. More recently, linkage to the South African National Cancer registry has also been completed [28]. Imputation of treatment start dates using the NHLS laboratory data creates a promising additional source of “big” data for linkage opportunities.

Our results must be considered in light of their limitations. First, despite the large database used, the method was tested in just two well-resourced cohorts with well-documented laboratory results. Though these cohorts do represent very diverse populations and geographic regions in South Africa, they may not represent all populations and regions in the national HIV program. Additionally, changes to ART initiation protocols or different practices in different countries would limit the generalizability of these findings; we encourage further validation in settings using different initiation protocols. Researchers recently showed the potental to impute ART start dates using laboratory-measured repeat viral loads in New York City, where (unlike South Africa) viral loads are assessed routinely both before and after initiation [29]. This example illustrates the broad potential of lab-based imputation as well as the necessity of tailoring imputation algorithms to local clinical protocols. Second, our estimates of PPV and sensitivity were computed in a patient population known to be HIV-infected. It is possible that implementing our approach in a population of patients with unknown HIV status would have a lower PPV due to the presence of false positives. However, the requirement of a CD4 count in the previous 12 months is expected to screen out HIV-uninfected patients and minimize false imputation of start dates. In populations including HIV-uninfected patients, NPV and sensitivity are expected to be much higher than reported here. Third, the trend towards decreasing time to initiation from workup by calendar year may require adjustment of the 21-day interval assumption by calendar period. Fourth, our identification of ART workup dates relied on the ability to identify a patient’s *first* ALT/HB, which requires longitudinal linkage of identifier in the national NHLS laboratory data are currently under way by members of this research team. Fifth, some patients may transfer out of the cohort facilities after their work up and initiate elsewhere. However, this implies that the high PPV demonstrated in the Hlabisa cohort (95%) is actually an underestimate and that true PPV may be even higher. Sixth, we may slightly underestimate PPV for late 2012 as some patients may have initiated ART after the end of the study period in December 2012 (Table S2). Lastly, if some laboratory tests were not captured into the cohort databases, then our estimates of sensitivity (82.6%, 88.2%) would also be underestimates of the true values.

## Conclusion

Chronic disease management in low-resource settings is typically informed by routine laboratory monitoring and guided by standardized clinical protocols. We have demonstrated that routine laboratory testing data can be used to impute information about clinical decision-making that may not be complete in patient charts or aggregate statistics. The ability to impute ART start dates using lab workups has concrete implications for program monitoring and patient care in the South African national HIV CCMT program. Our focus on one disease, one treatment decision, and one country imply that caution should be exercised in generalizing from our results to other diseases, clinical decisions, and settings. However, our results offer a proof of concept. We are optimistic that this analysis may illuminate other opportunities to leverage routinely collected laboratory data to improve the accuracy and completeness of clinical records and improve chronic disease management in low resource settings in South Africa and beyond.

## Additional file

Additional file 1: Table S1. Distributions of sensitivity and median days to initiation by clinic. **Table S2**. Sensitivity of imputed start date and median number of days to initiation by calendar year. **Table S3**. Effect of changing the “matching” interval between ART workup and known ART start date on sensitivity of imputation method in Hlabisa and RTC cohorts. (DOCX 18 kb)

## Abbreviations

AIDS: Acquired immune deficiency syndrome
AIDS: Acquired immune deficiency syndrome
ALT: Alanine transaminase
ART: Antiretroviral treatment
CCMT: Comprehensive care management and treatment
CDF: Cumulative density function
Hb: Hemoglobin
HIV: Human immunodeficiency virus
NHLS: National Health Laboratory Service
NPV: Negative predictive value
PMTCT: Prevention of mother-to-child transmission
PPV: Positive predictive value
RTC: Right to Care
SE: Sensitivity
SP: Specificity

## Footnotes

1 This adjustment factor follows directly from Bayes Theorem, which says that PPV = sensitivity * Pr(ART)/Pr(workup). For example: if 100 patients have a lab work-up at a given clinic, then we expect that 95.4 of these patients will initiate ART within six months. However, those 95.4 patients represent only the 82.6% of ART initiators who also had a workup. Dividing by 82.6% yields an estimate of 115 total ART initiators at the clinic.
