# Supplementary material for: Imputing HIV treatment start dates from routine laboratory data in South Africa: a validation study
Source: BMC Health Serv Res. 2017 Jan 17;17:41. doi: 10.1186/s12913-016-1940-2 (PMC5240407; doi:10.1186/s12913-016-1940-2)
Supplement: Additional file 1: Table S1. — Distributions of sensitivity and median days to initiation by clinic. Table S2. Sensitivity of imputed start date and median number of days to initiation by calendar year. Table S3. Effect of changing the “matching” interval between ART workup and known ART start date on sensitivity of imputation method in Hlabisa and RTC cohorts. (DOCX 18 kb) [file 12913_2016_1940_MOESM1_ESM.docx]

**Supplementary Table 1 (for appendix): distributions of sensitivity and median days to initiation by clinic**

| **Clinic** | **# of ART initiators** | **Sensitivity** | **Days from work-up to ART initiation** |
| --- | --- | --- | --- |
| *Hlabisa facilities* | | | |
| H8 | 1066 | 83.4% | 15 (6, 34) |
| H11 | 1171 | 82.3% | 21 (10, 43) |
| H14 | 1093 | 84.9% | 17 (9, 30) |
| H15 | 2231 | 85.9% | 17 (11, 29) |
| H16 | 1664 | 85.6% | 13 (6, 27) |
| H19 | 2286 | 77.4% | 12 (5, 28) |
| H21 | 1459 | 87.8% | 23 (14, 39) |
| H24 | 4190 | 85.4% | 14 (8, 27) |
| H_Other | 6606 | 79.0% | 17 (7, 33) |
| *Right to Care facilities* | | | |
| RTC1 | 5165 | 95.1% | 36 (17, 58) |
| RTC2 | 8842 | 94.0% | 36 (21, 58) |
| RTC3 | 2004 | 71.4% | 14 (6, 28) |
| RTC4 | 4332 | 79.8% | 16 (7, 35) |
| RTC5 | 8604 | 91.6% | 18 (9, 42) |
| RTC6 | 5026 | 60.6% | 22 (10, 42) |
| RTC7 | 17769 | 94.4% | 16 (6, 37) |
| RTC8 | 9184 | 96.5% | 21 (9, 36) |

Notes: H_Other includes all Hlabisa facilities with fewer than 1000 ART initiators.

**Supplementary Table 2 (for appendix): Sensitivity of imputed start date and median number of days to initiation by calendar year**

| **Year** | **RTC cohort** | | | **Hlabisa cohort** | | | |
| --- | --- | --- | --- | --- | --- | --- | --- |
|  | **# ART initiators** | **Sensitivity** | **Median days to ART** | **# ART initiators** | **Sensitivity** | **PPV** | **Median days to ART** |
| **2005** | 2756 | 92.6% | 35 (14, 63) | n/a | n/a | n/a | n/a |
| **2006** | 4912 | 92.0% | 34 (17, 63) | n/a | n/a | n/a | n/a |
| **2007** | 5700 | 87.3% | 30 (14, 56) | 1963 | 86.4% | 94.1% | 23 (12, 39) |
| **2008** | 7359 | 85.5% | 28 (14, 53) | 3467 | 82.2% | 92.3% | 21 (8, 38) |
| **2009** | 9278 | 88.0% | 21 (11, 42) | 3223 | 86.3% | 94.8% | 18 (9, 35) |
| **2010** | 9686 | 87.8% | 21 (8, 41) | 3723 | 86.9% | 96.9% | 18 (9, 34) |
| **2011** | 7379 | 88.5% | 18 (7, 41) | 4607 | 78.2% | 97.1% | 14 (7, 25) |
| **2012** | 6037 | 89.5% | 16 (7, 33) | 4783 | 79.6% | 97.9% | 10 (6, 21) |
| **2013** | 4720 | 86.5% | 13 (5, 28) | n/a | n/a | n/a | n/a |
| **2014** | 3287 | 88.4% | 8 (2, 24) | n/a | n/a | n/a | n/a |

Notes: PPV = Positive Predictive Value. Because laboratory data were not systematically collected in the RTC cohort for patients who presented for care but did not initiate ART, we were unable to calculate PPV in the RTC cohort.

**Supplementary table 3: Effect of changing the “matching” interval between ART workup and known ART start date on sensitivity of imputation method* in Hlabisa and RTC cohorts**

|  | **Sensitivity** | |
| --- | --- | --- |
| **Interval prior to known ART start** | **Hlabisa cohort** | **RTC cohort** |
| 1 month | 62.7% | 54.2% |
| 3 months | 80.1% | 82.2% |
| **6 months** | **82.6%** | **88.2%** |
| 9 months | 83.3% | 88.2% |

***** Matching interval = the maximum time between ART workup and known ART start date for the imputed start date to be considered a “match” to the known ART start date. The primary analysis set this interval at 6 months; here we show the effect changing this interval has on sensitivity of the imputation method.

Sensitivity = the proportion of those with an ART workup in the specified interval who truly initiated ART
